# Long-term Follow-up of Severe Eosinophilic Hepatitis: A Rare Presentation of Hypereosinophilic Syndrome

**DOI:** 10.5041/RMMJ.10373

**Published:** 2019-07-18

**Authors:** Halim Awadie, Johad Khoury, Yaniv Zohar, Afif Yaccob, Ella Veitsman, Tarek Saadi

**Affiliations:** 1 Liver Unit, Rambam Health Care Campus, Haifa, Israel; 2 Pulmonary Division, Lady Davis Carmel Medical Center, Haifa, Israel; 3 Pathology Institute, Rambam Health Care Campus, Haifa, Israel; 4 Bruce Rappaport Faculty of Medicine, Technion–Israel Institute of Technology, Haifa, Israel

**Keywords:** Autoimmune hepatitis, eosinophilic hepatitis, hepatitis, hypereosinophilic syndrome

## Abstract

Idiopathic hypereosinophilic syndrome (HES) is a rare, heterogeneous disorder characterized by a strikingly high eosinophil count (>1,500 cells/μL), over a long period of time (>6 months), with end organ damage. We present a 60-year-old patient with idiopathic HES with isolated liver involvement, a rare systemic disease and a rare solid organ involvement. The patient had a thorough investigational work up until HES was established, including liver biopsy. He needed intensive immunosuppressive treatment at first with steroids, then with azathioprine in conjunction with a low dose of steroids. After 16 years of follow-up, the patient showed no evidence of liver dysfunction. To the best of our knowledge, this is the longest follow-up for a patient with HES-associated chronic hepatitis. Our observation suggests that, with appropriate treatment, liver involvement in HES may be well controlled without deterioration to advanced liver failure.

## INTRODUCTION

Idiopathic hypereosinophilic syndrome (HES) represents a heterogeneous group of disorders with the common features of prolonged eosinophilia of more than 1,500 eosinophils per microliter (μL) of peripheral blood for at least 6 months, with signs or symptoms of organ system dysfunction, without detectable cause.^1^,^2^ However, treatment should not be withheld in patients with hypereosinophilia of less than 6 months’ duration who have evidence of end-organ damage.^3^ As noted at the National Institute of Health conference in 1982 by Fauci et al., multiple organ systems have been reported to be involved; bone marrow hypereosinophilia was common, but a more severe clinicopathological involvement was of the heart and nervous system, while hepatic involvement occurred with a prevalence of 32%.^1^

Chronic hepatitis associated with hypereosinophilia has been very rarely reported worldwide. It commonly affects young middle-age (40–50 years) women. The signs and symptoms are variable, predominantly presenting as malaise, fatigue, anorexia, jaundice, and hepatosplenomegaly.^4^,^5^ Spry et al. showed that patients with HES may survive for many years, ranging from 0.8 to 11.5 years, with mean survival of 4.4 years.^6^

## CASE PRESENTATION

A 60-year-old man presented to the emergency room with jaundice and elevated hepatocellular and cholestatic liver enzymes in accordance with hypereosinophilia. The patient denied fever, abdominal pain, nausea, or vomiting. He described a pale stool and dark urine. He also denied consuming any drugs, herbal medicine products, or alcohol; he reported no personal or family history of liver or biliary disease, blood transfusion, or exposure to toxins. He denied a history of allergy, asthma, or recent travel.

On physical examination, the patient was alert, with prominent jaundice. He had signs of chronic liver disease: spider angiomas, palmar erythema, and clubbing. He had no ascites, gynecomastia, or testicular atrophy.

Laboratory findings are summarized in Table 1. Complete blood count showed hypereosinophilia, 3,636 eosinophils of 12,000 leukocytes; hemoglobin and platelets were within the normal range. Creatinine, urea, and electrolytes were normal. Total and direct bilirubin on admission were 13.5 mg/dL and 12.1 mg/dL, respectively. Liver enzymes were elevated in a combined pattern (hepatocellular and cholestatic) (Table 1). International normalized ratio (INR) was slightly elevated at 1.44, and partial thromboplastin time (PTT) was normal. Stool tests were negative for parasites.

**Table 1:** Blood Tests on Presentation.

Test	Result (Normal Range)
WBC (×10^3^/μL)	12.0 (4.8–10.8)
Eosinophils (%)	30.3 (0–7)
HGB (g%)	15.8 (13.5–17.5)
PLTs (×103/μL)	257 (130–450)
Creatinine (mg/dL)	1.0 (0.4–1.3)
BUN (mg/dL)	8.1 (5–20)
Na (mmol/L)	137.0 (133–147)
K (mmol/L)	3.8 (3.5–5.3)
Amylase (U/L)	79 (30–140)
Bilirubin total (mg/dL)	13.5 (0.2–1.0)
Bilirubin direct (mg/dL)	12.1 (0–0.4)
AST (U/L)	236 (5–40)
ALT (U/L)	200 (5–40)
GGT (U/L)	634 (5–60)
ALP (U/L)	197 (30–115)
INR	1.44 (0.9–1.3)
PTT (s)	37.3 (27–38)

ALP, alkaline phosphatase; ALT, alanine transaminase; AST, aspartate transaminase; BUN, blood urea nitrogen; GGT, gamma-glutamyl transpeptidase; HGB, hemoglobin; INR, international normalized ratio; K, potassium; Na, sodium; PLTs, platelets; PTT, partial thromboplastin time; WBC, white blood cells.

Serologic tests were negative for the following: hepatitis B surface antigen; anti-hepatitis B core immunoglobulin M (IgM); anti-hepatitis A virus IgM; anti-hepatitis C virus antibody; anti-*Cytomegalovirus* IgM; anti-Epstein–Barr virus IgM; anti-nuclear antibodies; anti-smooth-muscle antibodies; anti-mitochondrial antibodies; and anti-neutrophil cytoplasmic antibodies. Serum ferritin and ceruloplasmin levels were also normal.

Abdominal ultrasound performed on admission revealed hepatomegaly of 18 cm, lobulated edge, but no lesions were seen within the liver. Gallbladder and bile ducts were normal. A hyperechogenic non-homogenized lesion was seen at the head of the pancreas, 5.2 cm in diameter, which was suspected to be a space-occupying lesion.

Chest and abdominal CT demonstrated a suspected space-occupying lesion at the head of the pancreas, while liver, gallbladder, and spleen were normal. After further workup in the Department of Surgery, an endoscopic retrograde cholangiopancreatography (ERCP) was performed and did not show any abnormality. Both cytology and serologic tumor markers were normal. The patient developed post-ERCP pancreatitis which was managed conservatively with no complications. Following thorough investigation, the pancreatic lesion was concluded to be benign, and clinical follow-up was advised.

An incidental finding of a suspicious thyroid nodule was seen in the chest computed tomography (CT). Fine-needle aspiration for the nodule confirmed the diagnosis of papillary carcinoma. The patient had an elective total thyroidectomy and thyroxin replacement therapy was initiated.

**Figure 1:**
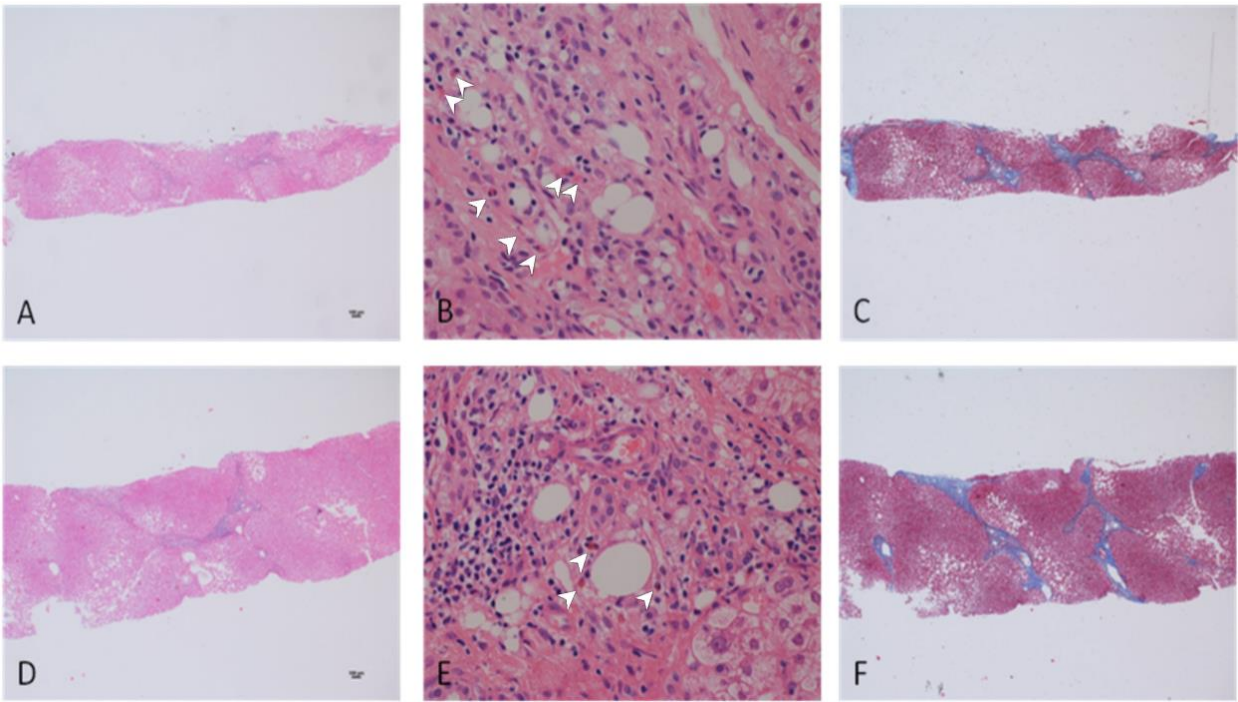
Liver Biopsy at First Evaluation and Repeated Biopsy Four Years Later. A liver needle biopsy taken within the first evaluation showed preserved lobular architecture (**A**, HE 4×) and mild portal inflammation with several eosinophils seen in the infiltrate (**B**, HE 40×). Masson trichrome stain showed focal bridging fibrosis (**C**, trichrome 4×). A second biopsy taken four years later showed portal inflammation (**D**, HE 4×) with fewer eosinophils in the infiltrates (**E**, HE 40×) and no change in fibrosis (**F**, trichrome 4×). White arrowheads indicate eosinophils.

At this point, a liver needle biopsy was performed (Figure 1, panels A–C), showing preserved lobular architecture (Figure 1A), mild portal inflammation with several eosinophils seen in the infiltrate (Figure 1B), focal bridging fibrosis (Figure 1C), and revealing features of chronic active hepatitis and marked eosinophilic infiltration with bridging fibrosis.

Bone marrow biopsy excluded lymphoproliferative disease, and a diagnosis of idiopathic hypereosinophilic syndrome with eosinophilic hepatitis was established. Following discharge, the patient attended our Liver Unit for follow-up.

At this stage, he remained asymptomatic with marked eosinophilia and abnormal liver function test. Despite being asymptomatic, and due to the risk of progressive liver function, treatment with systemic corticosteroids (prednisone 40 mg/d) was started. Three weeks later, a marked drop of eosinophilic count to a normal level was seen, in accordance with normal levels of bilirubin and normal hepatocellular enzymes, while the cholestatic liver enzymes remained elevated.

A slow tapering down of prednisone over 2 months was initiated; however, a rapid rise of peripheral eosinophilia (24%) was noticed when reaching the lowest dose of prednisone 10 mg/d, along with elevated liver enzymes and bilirubin. Following this, prednisone 40 mg/d was restarted, with improvement of eosinophilic counts in two weeks. However, minimal improvement of liver enzymes was seen during this time.

Maintenance therapy on corticosteroids was continued for several months with clinical and laboratory response. Due to the corticosteroids therapy, he developed diabetes mellitus, which necessitated insulin therapy.

Several attempts at systemic steroid tapering down resulted in recurrent liver enzymes elevations with the lowest effective dose of 10 mg. Thus, resuming prednisone 40 mg/d along with azathioprine 75 mg/d was started, with later slow tapering down of corticosteroids.

**Figure 2:**
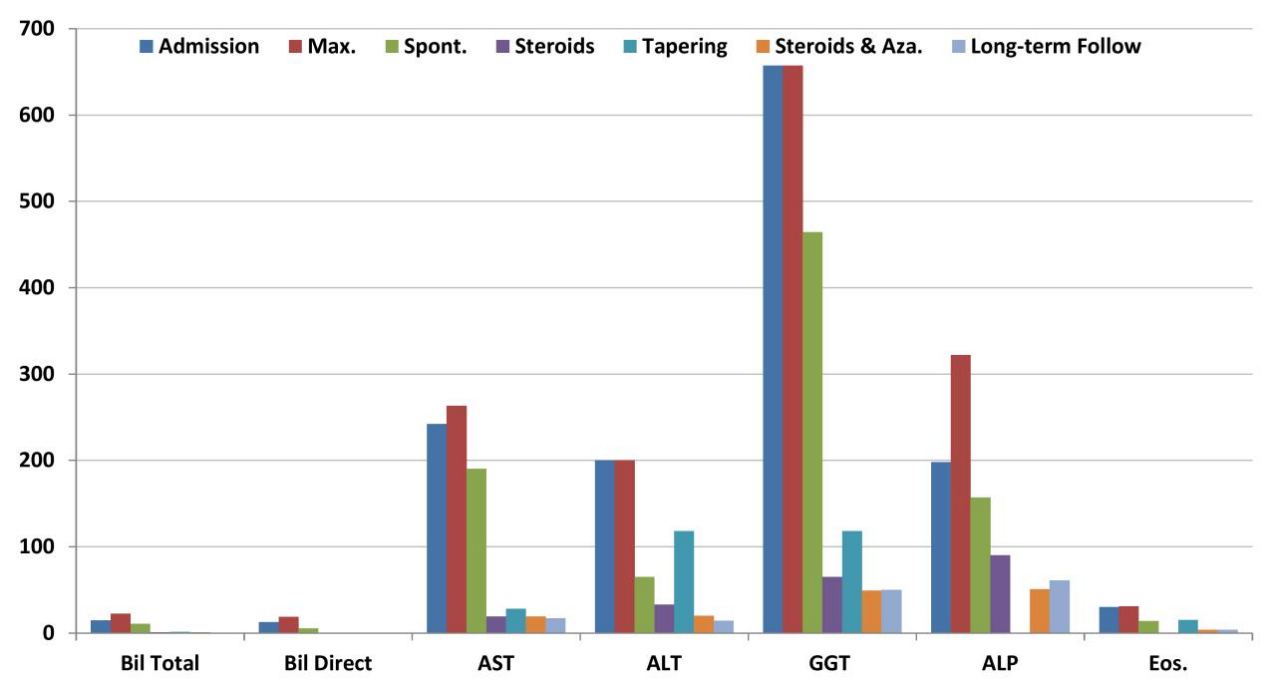
Eosinophilic Count, Liver Function Tests, and Enzymes from Presentation to Date. ALP, alkaline phosphatase (U/L); ALT, alanine transaminase (U/L); AST, aspartate transaminase (U/L); Bil Direct, bilirubin direct (mg/dL); Bil Total, bilirubin total (mg/dL); Eos, eosinophils (×10^3^/μL); GGT, gamma-glutamyl transpeptidase (U/L); Max, worst value during hospitalization; Spont, spontaneous improvement; Steroids & Aza, concomitant treatment of steroids an azathioprine; Tapering, tapering down of steroids.

Normal liver function study and eosinophilic count were achieved on prednisone 15 mg/d and azathioprine 75 mg/d (Figure 2). Further prednisone tapering down to the lowest effective dose of 5 mg/d and raising azathioprine to 100 mg/d was done.

Bilirubin and liver enzymes were normalized, as were albumin and INR. For histopathological assessment, a repeat liver biopsy (Figure 1, panels D–F) was done and showed portal inflammation (Figure 1D) with fewer eosinophils in the infiltrates (Figure 1E) and no change in fibrosis (Figure 1F, trichrome).

After 16 years of follow-up, the patient reported regular daily activity on chronic medications which included azathioprine 100 mg/d, prednisone 5 mg/d, thyroxin 150 μg/d, atenolol 25 mg/d, hydrochlorothiazide 12.5 mg/d, and ranitidine 40 mg/d.

## DISCUSSION

Idiopathic hypereosinophilia is of no special clinical relevance, except as a curiosity and diagnostic dilemma. However, when the eosinophilia is associated with organ system dysfunction that cannot be explained by another mechanism apart from the presence of eosinophils and the probability exists that the tissue damage causing the organ system dysfunction is directly or indirectly related to the eosinophils, then a frustrating and serious clinical problem arises,^1^ especially when patients present at a late stage in the disease.

In Western countries, the most common cause of eosinophilia is allergic reaction, particularly immediate hypersensitivity responses,^1^ and mostly affects young men in their fourth decades.^6^ In this case report, the patient presented with clinical manifestations of active hepatitis with elevated liver enzymes and hyperbilirubinemia along with peripheral hypereosinophilia. This entity of liver involvement in HES has been mainly described at autopsy and very rarely at a patient’s clinical presentation.

Over 16 years of follow-up, our patient did well generally, with episodic raising of his steroid dose on serologic flare and continuous azathioprine therapy. Liver needle biopsy did not show progression on chronic immunosuppressive therapy.

There are few reports in the literature of patients with histological features of chronic hepatitis and peripheral blood eosinophilia. The hypereosinophilic syndrome represents a heterogeneous group of disorders with the common features of eosinophilia of unknown cause for over 6 months and organ system dysfunction. According to Fauci et al., hepatic involvement was typified by hepatomegaly and minor abnormalities of liver function tests. The spectrum of pathologic findings included congested sinusoids, chronic hepatitis without cirrhosis, and peri-portal inflammation.^1^

Organ system involvement in idiopathic HES can vary and mainly includes hematological (100%) involvement. Other organs were reported to be involved, such as nervous system (64%), skin (56%), cardiovascular system (54%), lungs (40%), liver (32%), nose and sinuses (26%).^5^ In the bone marrow, findings ranged from benign eosinophilia to blast crisis, with a range of intermediate findings found in most patients.^1^ Several reports of HES have been described in case series, with few reporting liver involvement. Willocx et al. described the hypothesis of autoimmune processes in chronic active hepatitis, suggesting that protein release from damaged liver cells is followed by the production of autoimmune antibodies. Subsequent antigen–antibody interactions then presumably lead to further liver damage.^7^

Circulating immune complexes and elevated IgE levels are often present. Bone marrow involvement occurs in all patients, but heart and nervous system usually are most severely involved. Chemotherapeutic administration for lowering the eosinophil count results in marked improvement of the manifestations of HES, and functional improvement can occur. Without adequate treatment, the syndrome has a high morbidity and mortality and must be treated vigorously.^4^

Chronic hepatitis, hypereosinophilia, and autoimmune hemolytic anemia in one patient who was treated by longstanding systemic corticosteroids was described by Panush et al. Flare-ups of the hepatitis were reported twice without hemolysis or peripheral eosinophilia noted, suggesting that these abnormalities were more readily and continuously suppressed by small amounts of steroids than was the hepatic inflammation.^8^ The occurrence of these three processes together in one patient suggests that they may be related to abnormal host-immune responses.^8^

Foong et al. reported a case of HES and acute hepatitis that progressed to chronic hepatitis along with immunochemical evidence that implicated eosinophils as the primary etiological factor for liver damage. This patient was solely treated with corticosteroid therapy for maintenance for more than seven years, with clinical and laboratory response.^9^

Croffy et al. reported a case series of four patients in 1998 with HES and chronic hepatitis, all of whom were treated with prednisone as maintenance therapy and two with additional azathioprine. A diagnosis of autoimmune hepatitis was excluded in these patients on clinical and laboratory basis. In three of the four patients, there were mild histological changes in liver biopsy with moderate portal triad enlargement and moderate portal necrosis, with little fibrosis but without bridging fibrosis. To the best of our knowledge, including our case report, all patients were male and no other autoimmune disease was correlated with the diagnosis of HES and chronic active hepatitis.^4^

In our case, the patient responded well to corticosteroid therapy, with normal eosinophilic counts and normal liver function study and halting of the progression of hepatitis as documented in a liver histopathology specimen. Although the patient had a good response to systemic steroid, he developed diabetes mellitus that was uncontrolled on oral hypoglycemics and required subcutaneous insulin therapy. As a result of serious steroid adverse effects, an additional immunosuppressive therapy was initiated by azathioprine, while tapering down prednisone. The lowest effective dose of prednisone reached while on azathioprine 100 mg/d was 5 mg/d. Up to the time of this publication, after 16 years of follow-up, the patient is doing well on the aforementioned medication, without any complaints or adverse effects. To the best of our knowledge, this is the longest period of follow-up in chronic hepatitis caused by HES.

According to this case, eosinophilic hepatitis can be managed similarly to autoimmune hepatitis. We can start treatment with steroids and thereafter add a steroid-sparing immunomodulator such as thiopurine. It is not clear whether the prognosis of eosinophilic hepatitis is similar to autoimmune hepatitis.

In our case, aggressive treatment was started early to prevent severe complications, such as liver failure. This approach may be considered in similar cases.

## CONCLUSION

Hypereosinophilic syndrome can involve the liver and can cause severe hepatitis. Appropriate treatment with immunosuppressive drugs, as in autoimmune hepatitis, may help control liver damage and prevent progression of liver disease. This entity is rare and should be considered in patients presenting with hepatitis and eosinophilia. The long-term prognosis may be excellent if the disease is well controlled; however, conclusions cannot be made upon a case report, and larger trials or case series are needed.

## Abbreviations

CT: computed tomography
ERCP: endoscopic retrograde cholangio-pancreatography
HES: hypereosinophilic syndrome
IgM: immunoglobulin M
INR: international normalized ratio
PTT: partial thromboplastin time.
